# The biology of sexual development of *Plasmodium*: the design and implementation of transmission-blocking strategies

**DOI:** 10.1186/1475-2875-11-70

**Published:** 2012-03-16

**Authors:** Robert E Sinden, Richard Carter, Chris Drakeley, Didier Leroy

**Affiliations:** 1 The Jenner Institute, University of Oxford, Old Road Campus Research Building, Roosevelt Drive, Oxford OX3 7DQ, UK; 2 Institute of Immunology and Infection Research, University of Edinburgh, Edinburgh, UK; 3 Department of Immunology & Infection, London School of Hygiene and Tropical Medicine, London, UK; 4 The Medicines for Malaria Venture, Cointrin, Geneva, Switzerland

## Abstract

A meeting to discuss the latest developments in the biology of sexual development of *Plasmodium *and transmission-control was held April 5-6, 2011, in Bethesda, MD. The meeting was sponsored by the Bill & Melinda Gates Foundation and the National Institutes of Health, National Institute of Allergy and Infectious Diseases (NIH/NIAID) in response to the challenge issued at the Malaria Forum in October 2007 that the malaria community should re-engage with the objective of global eradication. The consequent rebalancing of research priorities has brought to the forefront of the research agenda the essential need to reduce parasite transmission. A key component of any transmission reduction strategy must be methods to attack the parasite as it passes from man to the mosquito (and vice versa). Such methods must be rationally based on a secure understanding of transmission from the molecular-, cellular-, population- to the evolutionary-levels. The meeting represented a first attempt to draw together scientists with expertise in these multiple layers of understanding to discuss the scientific foundations and resources that will be required to provide secure progress toward the design and successful implementation of effective interventions.

## Background

It is now recognized that understanding and attacking the parasites responsible for infection of the mosquitoes is critical to the international efforts striving to eliminate malaria [1]. Further, filling the gaps in the understanding of parasite sexual biology could greatly help the development of effective malaria transmission-blocking interventions such as, but not limited to, vaccines and drugs. In his introduction to the meeting Richard Carter said that since the 2007 call to eradicate malaria, the field has become both energized and focused to a degree not seen in 50 years, concluding that malaria transmission reduction is once again a priority on the research agenda.

The objectives of the meeting were to:

• Develop a comprehensive landscape of research initiatives, programmes, and funding vehicles in *Plasmodium *gametocyte biology,

• Identify and prioritize key gaps in the knowledge base and research agenda (see summary table of key deliverables Table 1).

**Table 1:** Summary of Key deliverables

***Key deliverables to understand transmission dynamics***	• Understanding of the low levels of natural transmission
	• Clarification of the relationships between the different methods of measuring transmission to the mosquito
	• Better definition of the infectious reservoir, and its role in control programmes
	• Biomarkers to distinguish infectious from non-infectious hosts
	• Improved integration of laboratory and field experimentation and data
***Key deliverables to understand gametocyte biology***	• New markers for commitment to gametocytogenesis
	• Improved methods for the purification of the different stages of sexual and sporogonic development.
	• Understanding of the pathways regulating sexual development (both gametocytogenesis and gametogenesis)
	• Understanding of parasite metabolism during sexual and sporogonic development
	• Improved understanding of the molecular basis of fertilization
	• Understanding of the mechanisms controlling gametocyte distribution in the host bloodstream.
***Key deliverables for translation***	i) **Vaccines**
	• Understand the biological relevance of membrane feeding assays
	• Confirm structural and immunological fidelity of both current and new candidates
	• New platforms to enhance and prolong antibody responses
	• Designs for new field studies in a variety of endemic settings to evaluate TBVs alone and in combination
	• Develop bifunctional vaccines to attack both population bottlenecks (e.g. ookinete and liver schizont)
	ii) **Drugs**
	• Phenotypic screens for novel entities against gametocytes and ookinetes
	• New target-based screens
	• Understanding of the mode of action of primaquine
	• Identification of dual activity compounds from the known library of 25,000 compounds with schizonticidal activity
	• Identification of novel transmission-blocking-specific compounds, to explore possible combinations with blood schizonticides
	• Methods for sustained drug delivery
	**iii) 'Out-of-the-box'**
	• Reagents to modulate the mosquito innate immune system
	• Understanding of the roles of natural-, or genetically modified- microflora in regulating malaria transmission in the mosquito.
***Key research tools required***	• Molecular markers for all stages of sexual development
	• Widespread availability of reagents for all 'genes' (GM parasites expressing tagged proteins or knockouts; monoclonal antibodies)
	• Improved access to high resolution, live imaging.
	• Publicly available archives of numerical and microscopic data
	• Wider access to transmission facilities
	• GM rodent parasites expressing key proteins/gene-products from human malaria parasites
	• Improved mathematical models of malaria transmission.

• Share and understand priorities of different institutions working in this research area.

In convening this meeting, the NIH/NIAID and the Bill and Melinda Gates Foundation (BMGF) created an opportunity for international researchers and stakeholders to share research updates, identify critical experiments, and discuss the resources required to advance these objectives. The key research questions addressed by separate sessions of the meeting included:

1. What are the critical factors comprising/regulating gametocyte biology?

2. What are the critical factors influencing transmission dynamics?

3. What are the best ways to target current and future interventions?

4. What are the critical needs for the field to advance?

## What are the critical factors comprising/regulating gametocyte biology?

In recognizing the meeting would discuss sexual development of all species of *Plasmodium*, it was highlighted that in a minority of parasite species (in the subgenus *Laverania*) e.g. *Plasmodium falciparum *sexual development differs substantially from the majority (in the subgenus *Plasmodium*) e.g. *Plasmodium vivax Plasmodium malariae, Plasmodium ovale, Plasmodium knowlesi *and *Plasmodium berghei*. The importance of recognizng the underlying molecular differences in developmental strategies between these species-groups is critical both to experimental analysis, and to the rational design and understanding of interventions in the clinical/field setting. For example, the protracted (9-12 day) developmental period of gametocytes in *P. falciparum *whilst offering a near unique opportunity to dissect gametocyte maturation at the molecular level, results in a totally different relationship between morbidity (and therefore presentation to the clinic) and transmission, i.e. while cases of vivax may be more infectious to mosquitoes prior to presentation at a clinic, falciparum cases are more infectious after presentation.

## How do sexual stages arise (gametocytogenesis)?

Pietro Alano discussed commitment to, and differentiation of, sexual stages at a cellular level. Pfs16 and Pfg27/25-the first molecular markers of gametocytes of the human malaria parasite *P. falciparum*, are expressed 24 hours into their development, Christian Doerig added that Pfnek-4 expression might also identify sexually committed gametocytes. It has been shown that all the progeny from a single blood stage schizont are already committed to become asexual parasites or either male or female gametocytes [2,3]. Until recently the community lacked markers to identify within a schizont which path its progeny will take, however Kim Williamson described a recent microarray analysis of *P. falciparum *which identified 11 genes up-regulated in committed parasites, three of which could be identified in 'asexual' schizonts. Christian Doerig noted that the normal excess of schizonts that are not committed to gametocytogenesis makes studying the biochemistry of the committed-few very challenging. Pietro Alano noted that recently has it been possible to purify to virtual homogeneity Stage-Ia gametocytes, thereby enabling differential proteomics [4].

Following these presentations Chris Drakeley commented that, while there is laboratory work indicating that 'stress' induces gametocytogenesis, as posited by the early studies of Sinton [5], in natural infections there seems to be a constitutive commitment to sexual development which itself may be modulated by extraneous factors. Factors reportedly influencing gametocyte development included host RBC age, hypoxia and exposure to schizonticidal drugs. Notwithstanding the comment that recent work in vitro stems from just one or two strains of laboratory-adapted parasites, data presented by Kim Williamson suggested in vitro gametocyte conversion of *P. falciparum *is consistent with a constitutive 10% rate of induction. She too recognized that conversion rates are more complex in the field, where parasitaemia alone does not predict gametocyte production, and as suggested by the early studies, she surmised the onset of clinical symptoms, if they occur, is a better predictor.

Sarah Reece offered an evolutionary ecology perspective on malaria gametocytogenesis emphasizing the need for its better analysis in order to understand malaria transmission. For example, she suggested the conventional view that malaria transmission will cease when basic reproductive rate (Ro) is reduced to less than one (see below) may not hold. Under transmission reducing measures, theory suggests parasites may adjust their gametocyte sex ratio to maximize their reproduction and thereby raise Ro again [6]. Because sex allocation rules fit very diverse taxa [6], there is hope that knowledge on sex allocation obtained from the study of rodent malarias in the laboratory will be relevant to human malaria transmission.

## How do gametocytes develop and differentiate and get ready for the flight?

Pietro Alano reported that kinase knockouts (KO's) do not affect gametocyte production. Compared with merozoites, he noted that young gametocytes have a very high level of expression of export-related proteins [7], that erythrocytes containing young gametocytes have a very low antigenic profile, and knobs- structures that are associated with sequestration of asexual blood stages (ABS), are not displayed on the surfaces of the gametocyte-infected red blood cell (giRBC). Immature gametocytes of *P. falciparum *do nevertheless sequester and there is a need to identify the parasite molecules involved.

Bob Sinden discussed the cellular organization of mature gametocytes. Mature gametocytes of both sexes while sensitive to inhibitors of respiration, are less sensitive to anti-metabolites and schizonticides than the immature forms and asexual stage parasites [8-10]. Mature male and female gametocytes differ markedly at both the molecular and cellular levels. The female is a typical eukaryotic egg cell having a well-developed endoplasmic reticulum, mitochondrion and apicoplast in preparation for rapid development as a zygote. This rapid development is mediated in part by DOZI-regulated [11] translationally repressed mRNA species, encoding 'early translated proteins' such as P28, these messengers are located in discrete foci in the cytoplasm [12]. The mature male gametocyte, by contrast, has a markedly reduced ribosome and endoplasmic reticulum network; it will contribute only plasma membrane, nucleus and an axoneme to the male gametes and zygotes. Osmiophilic bodies are found in both male and female gametocytes but are more abundant in the latter; these secretory organelles are involved in the parasites' escape from the red blood cell (RBC) during gametogenesis [13,14].

Kim Williamson further discussed the biochemistry and gene expression of gametocytes. As indicated above, protein synthesis and degradation are essential for gametocyte maturation. The Alamar Blue viability stain confirms that gametocytes are metabolically active and can be targeted with antimetabolites e.g. epoxomicin and cycloheximide [15]. Work is in progress on assays to identify essential metabolic pathways in gametocyte maturation; it has been shown that pyrimidine biosynthesis is not required. It was suggested a more profound understanding of the variations in metabolism between the different sexual stages (immature/mature; male/female; gametocyte/gamete) is needed to provide a secure appreciation of the observed inability of the current anti-malarial portfolio to inhibit malaria transmission stages.

## What happens during gametogenesis?

Oliver Billker inferred gametogenesis is optimized for speed not quality of outcome. From the moment of their induction to undergo gametogenesis, gametocytes of both sexes take around ten minutes to complete "rounding up" and emergence from the RBC, simultaneously the male undergoes "exflagellation" to release the 8 male gametes. Induction is triggered by a drop in temperature, an increase in intracellular pH, and exposure to the mosquito exflagellation factor xanthurenic acid (XA) in the mosquito blood meal [16]. Within the gametocytes, kinases are involved in the regulatory activation pathways [17]. Reverse genetics has been particularly helpful to dissect regulatory pathways in gametocytes - there are good phenotypes and phenotypic assays - but improvements in this area are needed. There is a project at the Sanger Institute, UK, to scale up reverse genetic approaches to provide a large library of gene-targeting vectors for *P. berghei*. Questions that remain unanswered include: "What is the origin of XA in the blood meal?", and "Can one activate gametocytes in the blood stream to eliminate their transmission potential?"

Nirbhay Kumar discussed cellular events and molecules in gametogenesis. To achieve this inside a mosquito blood meal, the parasite must adapt to a new pH-, temperature-, ionic/nutritional- environments, and to the components of the vertebrate and mosquito hosts immune systems to which the parasites are exposed. Potassium (K+) channels could play a role as possible drug targets. Knockout of the encoding gene (*pfkch1*) leads to loss of gametocyte infectivity in the mosquito [18].

Gene expression and gene control in gametogenesis was addressed by David Baker. Little is known in this area, beyond the key role of translation control mechanisms. The radically different environments of the host blood circulation, where the mature gametocytes exist in a quiescent state and that in the mosquito blood meal, present opportunities for the parasite to switch the pattern of gene expression. But to what extent does this take place? Do the environmental cues connect with signalling pathways leading to changes in gene expression? He reported that initial microarray analysis suggests there is a global regulation of both stage-specific transcription [19], and of steady state RNA levels.

## What happens during fertilization and after?

Andy Waters discussed the rapidly-completed cellular and molecular events of fertilization, which occur in the hostile environment of the blood meal. Male microgamete motility is fuelled by glycolysis, which is druggable [20]. In fertilization, a family of proteins posessing 6-Cysteine (6-Cys) domains (P45/48, P47, and P230) are expressed on the surfaces of gametes of both sexes; there is evidence that they are involved in fertility. 6-Cys KO's result in the production of very few ookinetes in vitro, and cause a 20-50 fold reduction in the parasites' transmissibility to mosquitoes. The female gametocyte transcriptome is overwhelmingly silenced in the RNA helicase-DOZI (Development Of Zygote Inhibited) KO [11], but protein translation is rapidly resumed during gametogenesis. In recognizing the common basis for motility of the merozoites, sporozoites and ookinetes, he suggested ookinete motility could be a target for multistage drug development.

Dietlind Gerloff described structural modelling of the gamete surface protein P230, an *in silico *activity [21,22] that has complemented the crystallographic studies on the ookinete surface protein P25 [23]. In 2005, the first three dimensional structure was produced for P230, suggesting its structural similarity to the *Toxoplasma gondii *surface protein SAG1 [21]. The domain structure of P230 is specific to apicomplexan parasites; the size of a single domain in this protein and its 6-Cys relatives was similar to that of the immunoglobulin fold. It was suggested the value of the structural models is that they can guide further experiments with these molecules and refine understanding of their interactions.

The genetics of fertilization and of the interactions of malaria parasites with mosquitoes was addressed by Lisa Randford-Cartwright. Both male and female gametocytes can be generated from a single haploid clone, sex is not, therefore, determined by sex specific chromosomes. Gametes of the same clone both self-fertilize (forming homozygotes) or cross-fertilize (forming heterozygotes) with equal frequency. Mating appears to be random between gametes of different genotypes [24,25] the resulting zygote is briefly diploid before the nucleus undergoes meiosis [26]. From laboratory crossing experiments, there is no evidence for mating types within gender, i.e. preferential mating, or exclusion of mating, between parasites of specific genotypes. When investigating natural malaria transmission people are generally found to be infected with at least two genetically distinct clones of malaria parasite of a given species. In wild-caught mosquitoes both homozygous and heterozygous oocysts have been observed [27] confirming that both selfing and crossing happen in the field. In laboratory experiments, vector efficiency is frequently higher with co-indigenous strains of *Plasmodium *and mosquito [28]. Linkage/quantitative trait locus (QTL) analysis is currently being employed to look at the genetic basis of such parasite strain/vector species specificity.

Bill Snell discussed fertilization in protists. The free-living protist, *Chlamydomonas*, appears to be a useful model for fertilization in *Plasmodium*. It is easy to grow, easy to manipulate, has well-characterized biology, and can be manipulated by the introduction of transgenes, but current evidence suggests this rarely occurs by homologous recombination [29]. Hap2 was discovered to be important for membrane fusion during fertilization in both *Chlamydomonas *and *Plasmodium *[30]. Hap2 is a single pass transmembrane protein that is absent in vertebrates but is widely conserved in plants, bees, *Trypanosoma, Toxoplasma*, and *Eimeria*. The fusion mechanism is likely to be conserved across these groups of organisms; on the other hand the initial coupling of cells is expected to be achieved by species-specific proteins.

## What are the critical factors influencing transmission dynamics?

The main objective of this session was to describe what is known about the dynamics of natural infectivity and the human reservoir of infection, and subsequently to identify some of the knowledge gaps related to the fundamental basic biology of gametocytes. It was recognized that malaria transmission is focal, i.e. it occurs within spatially limited areas - each focus of transmission is determined by the locations of vector mosquito breeding sites and the flight patterns of the resident anopheline vectors responsible for parasite dissemination. As defined by the Ross/MacDonald equation, malaria transmission is dependent upon many different, and targetable, biological events, which must be considered in concert and not in isolation. If the malaria case multiplication rate, Ro, is greater than 1, malaria transmission will be sustained within each focus. If Ro consistently falls below 1, the focus will cease to sustain transmission. As a very rough approximation, it was hypothesized any single intervention may be expected to reduce Ro by two- to four-fold when randomly applied (Saul, A, pers comm.; Carter R, unpublished). It was suggested targeting multiple interventions to sites of high transmission may increase their impact by a further two- to four-fold. By combining different interventions, powerful overall reductions in malaria transmission may be achieved if, and when, the reductions due to the individual interventions multiply together. Within a focus of transmission, malaria case incidence rates vary markedly across distances as short as a few tens of meters, and between foci malaria transmission rates can vary by orders of magnitude across distances of a few hundred metres to kilometres [31-33], distances that lie well within the flight distance of the mosquito. Because of this spatial heterogeneity in transmission even within a 'focus', transmission-blocking measures are predicted to be most effective when targeted to the specific locations of highest transmission intensity [31,34].

Recent descriptions of the basic biology of *P. falciparum *have, of necessity, relied on data generated from a limited number of laboratory strains in highly controlled experimental conditions. Data from the first half of the 20^th ^century [35] suggest the kinetics of gametocyte production, carriage and infectivity in diverse hosts living in areas of different malaria endemicity is likely to be much more complex than observed in vitro. Understanding some these complexities may lead to the discovery of new subtle and species-specific adaptations that facilitate transmission. Chris Drakeley provided an overview of the current epidemiological data related to *P. falciparum *gametocyte carriage and infectivity. He suggested one of the most important recent field observations is the confirmation of prior 'malaria-therapy' data [36] that high/sustained levels of gametocyte carriage can be frequently observed. These new field observations are based on sensitive molecular amplification techniques and show that in several in vivo anti-malarial treatment studies the prevalence of gametocytes on enrolment is in the order of 80-90% compared with the 15-30% measured by microscopy [37]. This might suggest that gametocytogenesis occurs much earlier in the infection than commonly thought and that the classic 'stress' phenomena perceived to be essential for in vitro gametocytogenesis (see above) may be different to those acting in vivo. Importantly these sub -patent/microscopic gametocyte densities are infectious to mosquitoes [38]. They invariably result in low prevalence and intensity of mosquito infection, but this may be 'offset' by the host being infectious for longer than previously assumed, for example in chronic infections in semi-immune adults.

Recognizing the numerous extraneous factors that regulate the infectivity of mature gametocytes [39], the relationship between gametocyte density and infectivity is unpredictable, especially in the case of *P. vivax *in SE Asia where a higher proportion of patients are infectious compared to *P. falciparum *(Jetsummon Sattabongkot). Similar data were observed in South America (Jo Vinetz) with a variety of parasite and host factors incriminated, including immune and metabolic mediators, of these the impact of sexual schizogony on *P. vivax *gametocyte infectivity has been particularly well-documented [40]. The role of the relative susceptibility of in- and out-bred vectors as modulators of transmission was also raised as an additional yet unknown variable.

The session continued with detailed presentations on the interactions between gametocytes and their human hosts (Teun Bousema). Little is known about the ligands that mediate interactions between the gametocyte-infected RBC (giRBC) and the human host, though there are several candidates such as the STEVOR and the RIFIN families of exported proteins. Gambian children showed antibody responses to the surface of late stage gametocyte-infected erythrocytes independent of asexual parasitaemia and these responses correlated with reduced gametocyte carriage (Colin Sutherland). The field currently lacks both assays to evaluate *P. falciparum *gametocyte sequestration, and specific reagents to investigate these phenomena. The identification of biomarkers to identify persons harbouring infectious gametocytes was considered paramount. Using data obtained from a flow-based technique to examine the deformability of gametocytes at different stages of development, Pierre Buffet and Catharine Lavazec described interactions between the giRBC and the spleen - a site implicated in gametocyte sequestration.

It was noted that little has been done to look at the co-evolution of human and parasite genetics in relation to infectivity to the vector. It was muted that the human genotypes influencing the development of asexual parasites may directly or indirectly modulate transmission (David Modiano), examples of such interactions have been documented previously [39].

The practical implications of targeting gametocytes and infectious individuals in intervention campaigns were presented. Lucy Okell proposed that mass drug administration will have most success in reducing transmission. The practicality of targeted approaches depends on the sensitivity of the diagnostic to identify parasite carriers. Recognizing that a majority of infected persons are gametocyte carriers, it was asked whether simple, more robust and sensitive methods to detect asexual parasites might provide a more cost effective approach to the identification of potentially infectious individuals. Geoff. Targett and Gillian Stresman highlighted that many of the problems in targeting interventions to the appropriate population are shared with other control approaches, notably how one identifies the individuals to be targeted in both short-term and stable foci, in hotspots within these foci, and in travellers? In the absence of a transmission-blocking vaccine (TBV), current active transmission reduction approaches rely on drugs. There remain issues as to the best anti-malarials to use, primaquine currently being the only available gametocytocidal compound. The effect of artemisinin combination therapy (ACT) on transmission may be over-estimated because despite reducing gametocyte carriage [41] the few gametocytes that remain after treatment, as found with chloroquine treatment [42], can be very infectious (Abdoulaye Djimde).

## What are the best ways to target current and future interventions?

Recognizing the focus of the malaria eradication agenda has now expanded from the previous focussed objective of reducing morbidity and mortality in the human host, to protection against neo-infections, it is necessary to embrace the key target of transmission reduction. An expanded array of interventions must therefore be envisaged. These may include measures to either inactivate or eliminate parasites in both the human host *and the mosquito vector*, with for example drugs, vaccines, biological control (fungal or bacterial pathogens of mosquitoes) or genetic strategies to reduce mosquito populations or their susceptibility to *Plasmodium*.

Patrick Duffy reviewed the current interest in TBV. Rational development of TBVs requires that putative target proteins, on the surface of extracellular parasites in the mosquito vector, or components of obligate pathways and interactions, are better characterized by 'omics' approaches. To date, 24 proteins have been described as being inducers of potential transmission-blocking antibodies. Of the current lead candidates, two are expressed on the surface of gametes and in the intracellular compartment of gametocytes (P48/45 and P230), two others, P25 and P28 on the surface of the ookinetes. While producing protein antigens of appropriate immunogenicity remains challenging, it was suggested that solutions do exist e.g. *Pseudomonas aeruginosa *exo-protein A (EPA) [43] and *Corynebacterium diphteriae *cross-reactive material 197 (CRM197) [44] have been proven effective delivery mechanisms.

The question of deciding on the right target product profile for a TBV needs to be revisited carefully and should include integration of vaccines with other interventions. Identifying the target population for a (long-lived) TBV may be more straightforward than for a (short-lived) drug, Dr Duffy suggested every infected individual should be considered to be capable of transmitting the disease and therefore everyone in the area of transmission should be immunized with a TBV. This is in marked contrast to existing vaccine delivery systems in malaria endemic regions which target infants. There is now a trend toward performing Phase-1 trials in endemic settings. Considering that malaria transmission is focal, understanding micro-epidemiological transmission patterns in such settings is an essential pre-requisite to the determination of which geographic foci within the community should be preferentially targeted.

Didier Leroy reviewed the agenda for discovery of transmission-blocking drugs (TBD). The identification and validation of new potential drug targets are two highly challenging and time consuming processes whatever disease is considered. To date, screening of asexual blood stages of *P. falciparum *has been much more successful than molecular target-based approaches and has generated up to 25,000 hits with potencies below 1.5 μM. This is now being complemented effectively with work on a limited number of genetically validated molecular targets with essential roles in parasite development e.g. the protein kinases PfPKG, and Pfnek2 which potentially play key roles in the formation of gametes and ookinetes respectively. Similarly PfPK7 - essential for oocyst maturation in the mosquito midgut, might be targeted by long-lasting drugs, these might prove to be challenging to develop (see below). Despite the arsenal of new generation high tech assays and readouts, no medium/high throughput solution is currently available to screen sporonticidal compounds. It is urgent that the generation of 'omics' data is completed to improve understanding of the molecular regulation of sexual development, e.g. in late gametocytes, the digestion of haemoglobin is turned off, and 'simultaneously' mitochondrial function and the tricarboxylic acid (TCA) cycle are turned on.

In attempting to reduce Ro, gametocytes, and particularly their mature forms (stage V), may prove to be key targets for transmission-blocking agents. Clinical studies have shown primaquine efficiently eliminates gametocytes [45], notably when combined with sulphadoxine-pyrimethamine and artesunate [46]; the pathways, metabolites and molecular targets of primaquine remain to be identified. It can reasonably be hypothesized that elucidating the mode of action of primaquine against gametocytes would pave the way for the discovery of new anti-malarials.

Today, better assays are needed to investigate the downstream impact that drugs, administered to the gametocyte in the human host, might have on development within the mosquito vector and transmission. From a drug development perspective such drugs are attractive because of the comparative simplicity of drug delivery. By contrast the design of drugs specifically targeting the ookinete requires that the compound has a half life that matches that of the mature gametocytes. While this is readily achieved in the majority of malaria species (where gametocytes have a very short 1-2 day half life), it is more difficult with *P. falciparum *(where mature gametocytes can survive for up to three weeks). Targeting the oocyst with drugs (or vaccines) is even more problematic, because the deliverability and exposure of oocysts to drugs is impossible to control, potentially leading to the risk of accelerated selection of resistance.

Considering the current level of knowledge of anti-malarials targeting the asexual parasites in human blood and the constraints of drug development, one ongoing strategy to identify transmission-blocking drugs is to screen known blood schizonticides against late stage gametocytes. Today, the possibility of pursuing a drug development programme on a molecule that would block transmission but wouldn't cure the disease in the treated host is also recognized as a valid and valuable addition to our armamentarium. This will necessitate unbiased screening of large compound libraries against gametocytes and ookinetes. A novel concept of using drugs to induce gametogenesis in the human host was suggested. This has a two-fold attraction: first, the treated parasite will be incapable of transmission and second, the formation of gametes in the human host would require the expression of known potent transmission-blocking (neo) antigens (such as P25, P28) thus enhancing the impact of natural transmission-blocking immunity. Recognizing that, naturally, > 99% of gametocytes will die within the human host and thus present antigens to the immune system, it was appreciated that responses to gametocyte antigens per se (e.g. P230, P48/45) would not be significantly enhanced.

Carolina Barillas-Mury reviewed interventions targeting the parasite in the vector; she emphasized past wisdom that the only malaria control strategies that have been successful to date have included a component that controls the vector. The mosquito's immune system lacks an adaptive immune response arm and, therefore, no malaria-specific immune response awaits a subsequent infection. Recognizing the innate immune response is the only protection the mosquito can rely on to fight against the parasite, an interesting feature is the ability of the IMPer/Duox pathway to regulate the response when the midgut is invaded by parasites or bacteria. When this system is silenced/inhibited, the mosquito's midgut immune system efficiently reduces oocyst numbers. This interesting regulatory pathway needs to be further investigated and the possibility to inactivate this system specifically in mosquitoes, either directly with insecticides or indirectly with a partner drug in a combination therapy, has to be assessed. It would be interesting to know whether other similar pathways might be open to attack.

## What are the critical needs for the field to advance?

The following synopsis divides the conclusions reached into two distinct areas: firstly outstanding questions concerning the biology of the sexual stages of malarial parasites that are considered to be too fascinating and fundamentally important to our understanding of life processes to be overlooked, irrespective of whether they may have potential application; and secondly those areas where it is essential to develop a greater understanding to discover, develop and apply effectively interventions targeted to the sexual stages. It was clear to many participants that all too obvious opportunities for potent interventions against transmission of *Plasmodium *to the vector have been neglected for ~40 yrs. It might be anticipated that the substantial body of available knowledge accrued in this period of neglect might rapidly be converted into new and effective interventions.

## Fundamental biology of sexual development

### Induction of sexual development

The basic questions as to how the developmental decision between asexual and sexual development is induced and regulated remain at the forefront of the key unknowns. Fundamental issues as to whether the switch is constitutive and/or subject to qualitative or quantitative 'environmental regulation' remain unresolved and contested. Investigators must be prepared to ask whether this regulation differs between the parasite species. Whilst it is clear that in some species merozoites released from the pre-erythrocytic schizont can be committed to the sexual pathway (as it is in many 'primitive' haemoproteids) [47], it remains unclear as to whether, following RBC infection, there may be specific environments in the vertebrate host where induction is more common. Recent Piggy-Bac mutagenic studies on *P. falciparum *have identified for the first time genes essential for gametogenesis that are detected in cells that morphologically cannot be identified as gametocytes; this could be key to the identification of a switch described in the preceding sexually committed asexual schizont.

### The genetic regulation and molecular mechanisms of sexual differentiation

Although this field is developing rapidly the molecular basis underpinning the morphological, metabolic and functional maturation of gametocytes is poorly understood. The potential power of systematic 'omics' technologies remains compromised by the large number ~50% of the malarial genes that remain un-annotated. Data generated to date have provided a rational explanation for the induction and specificity of natural immunity to gametocyte antigens. The fascination of understanding the genetic and 'environmental' control of sexual development; the roles of genetic master-regulators' e.g. AP2, and DOZI; the roles of individual genes; of the design, assembly and functions of molecular machines and organelles was apparent to all. Nowhere is this curiosity more challenged than in the 'explosive' process of male gamete formation, where rates/mechanisms of DNA replication and axoneme assembly challenge any current understanding. From, and for, such studies improved markers of the numerous stages of sexual development must emerge.

Probably the biggest gap in the understanding of gametocyte maturation is the molecular description of gametocyte metabolism. Inhibitor/drug studies suggest significant differences between the immature (trophozoite-like) and morphologically mature (arrested in Go) gametocytes (protein synthesis being down-regulated in the latter). Mitochondrial/energy production pathways throughout sexual development and early sporogonic development may provide a rich vein to be mined. Such studies will benefit from standardized culture methodologies, and would provide one route to the development of effective drugs to block transmission.

### The biology of sexual development in the infected host and mosquito vector

There was considerable debate as to the molecular basis for the sequestration of immature *P. falciparum *gametocytes, and the need for appropriate markers/tools to study the phenomenon, the relevance of this topic, if any, to any parasite species outside the subgenus *Laverania *whilst unclear, was not discussed.

Clearly despite significant recent advances, the molecular basis of gamete-gamete recognition, gamete fusion and onward zygote development remains poorly understood. The parallel influences of immunological drivers of polymorphism in gamete surface proteins and the evolution of species-specific mechanisms of fertilization/zygote survival proffer fertile ground for theorists. Whilst recognizing the 'dominant' role of the large female gamete it is appropriate to ask what roles the incredibly simple male gamete plays in development e.g. determination of the polarity and subcellular coordination of the assembly and functions of the zygote/ookinete.

The complexity of malaria transmission through endemic populations was evident, key issues raised (but not fully understood) include differential transmissibility of parasite species, mixed species infections and genotypes; differential susceptibility of vector species and genotypes; the impact of human genotype; focality of transmission and intensity of transmission. Not least amongst the theoretical and practical questions raised was how these variables contribute to the definition of the reservoir of infection.

### Translation from knowledge to intervention

The necessity of understanding the impact of spatial and temporal distributions of infectious persons in modelling malaria transmission, and tailoring the design of intervention campaigns to areas of differing endemicity is clear, but recognizing the high prevalence of gametocyte carriage in infected persons the meeting was asked whether, during the implementation of an intervention campaign it is necessary to distinguish infectious from infected persons? The method by which infections are detected (active vs. passive case detection) remains critical, because the contribution of asymptomatic persons to the infectious reservoirs needs to be understood for an appropriate plan of intervention to be designed in each locality. If the operational model in the field remains either mass drug administration (MDA), or the detection and treatment of all malaria-infected individuals, every infected person will merit treatment to prevent transmission. The combined formulation of transmission-blocking compounds with schizonticidal drugs obviously addresses this need, and has been shown to be a most effective intervention strategy [48].

The potential of vaccines with transmission-blocking potential undoubtedly has a key role to play in any elimination/eradication agenda, whether in the treatment of focal areas of transmission or more broadly. All else being equal (immunogenicity; antigen diversity; longevity of effective response etc.) the theoretical advantage of a vaccine that might be boosted by any subsequent infection is clear, but still requires experimental validation. It was recognized that there would be significant advantage if the delivery of a transmission-blocking drug could be engineered to emulate that of a vaccine (prolonged delivery from a depot). It was also recognized that despite notable individual successes the discovery and development of both transmission-blocking vaccine and drug targets have to-date lacked rational genome-based discovery and prioritization.

A review of current progress in TBV development (Ashley Birkett) emphasized the current TPP is for a protein vaccine with a non-controversial adjuvant, which, if administered in no more than three doses within a six-month period would result in 85% blockade of mosquito infection intensity. Current focus is on Pfs48/45; Pfs25 and the mosquito antigen APN1. Recognizing that other candidates are under active investigation [49,50] it would be reassuring if it could be shown unequivocally that these are the best possible candidates. It was recognized that future needs must include research into vaccines targeted to *P. vivax*, and agreement on the development pathway, including standardized/harmonized assays of vaccine efficacy.

The objectives of development of new anti-malarials has been broadened, schizonticide discovery/development has been complemented by the clear recognition of the need to inhibit malaria transmission. Whilst acknowledging that early delivery of effective schizonticides will have a significant impact upon gametocyte production, and therefore transmission, two biological transmission targets have been prioritized: the hypnozoite (*P. vivax*; *P. ovale*); and sexual development (all species), for which the TPP is for fast-acting, long-lived drugs. It is recognized that the very small number of parasites at these critical phases of development theoretically offers the promise of slow selection of resistance, a problem that has blighted the development of the current generation of schizonticides. Such drugs might usefully complement diverse intervention strategies.

'Out-of-the-box' (i.e. non-drug, non-vaccine) thinking reminded the attendees of the now-proven potential of GM vectors, and para-transgenic approaches using mosquito symbionts/pathogens to express anti-parasitic moieties. The group highlighted the recent observations as to how the mosquito midgut microflora moderates the innate immune response and malaria infections, and raised interesting questions as to the indirect impact of current/future interventions upon the balances within this particular ecosystem. Significant thought will be needed to determine how these methods might be applied effectively in field situations.

## Tools needed to respond to the challenges ahead

### The molecular level

Many participants highlighted the need for powerful markers of expression of all sexual-stage genes. The value of expanded repositories of research tools e.g. gene knockouts, tagged proteins, monoclonal antibodies and the wider availability of high resolution in vivo imaging technologies cannot be overemphasized. With these improved tools it will be possible for community-wide action to analyse the function/impact of sexual stage genes, their chemical biology, and undertake HTP immunological studies on parasite biology. All HTP methods of parasite protein analysis remain critically dependent upon improved genome annotations of all relevant organisms, without this the recognized need for the discovery of the best vaccine, drug and other targets cannot be undertaken rationally or effectively.

### The experimental level

The contrast between the experimental tractability of the rodent malaria parasites (notably *P. berghei*) and the parasites of man was noted. In particular it was recognized that some of the tools currently used to determine the impact of transmission-blocking strategies on the human parasites are slow, expensive and available to few groups, e.g. Cat III culture facilities and secure insectaries. Even more exclusive were facilities to work on primate hosts with the consequence that abilities to study the transmission of *P. vivax*, or *P. knowlesi *are rare, and of the remaining species (*P. ovale, P. malariae*) or of mixed-species infections almost non-existent. The challenge of developing new safe and widely useable (preferably at the HTP level) analytic methods must be addressed. An area of immediate need is the requirement to understand the correlation between the various assays used to report the impact of transmission-blocking vaccines.

One proven, albeit still low throughput, approach to some of the problems above has been the production of transgenic rodent malarial parasites in which the relevant endogenous gene of interest has been replaced by the homologue from the human parasite. Where such exchanges are not complicated by fine molecular incompatibilities, these have a unique additional benefit in that the organism may not infect man thus removing major cost and security limitations to their study.

### The population level

Outstanding questions of transmission biology that confound the development of rational mathematical models include first, the need to understand heterogeneities at all levels of biology, from the gene through the biologies of the interacting organisms, of inter-personal infectiousness to the vector, to the spatial and environmental variations in parasite distributions in endemic communities. A second requirement is the need to recognize and define the recently recognized non-linearities in parasite dynamics [51,52] as they pass through the sequential phases of their life cycles, noting that these relationships may be subject to significant species-specific variations in host, parasite or vector.

One outstanding question that impacts upon the design of any effective transmission-blocking intervention is how to design and conduct appropriate field trials. Whilst this has been addressed in significant depth by other meetings [53] it is clear that diverse views remain. It might emerge that the design of trials of (transmission-blocking) bed nets could act as a secure framework for these deliberations.

## Competing interests

The authors declare that they have no competing interests.
